# Validation of the oral health behavior questionnaire for adolescents based on the health belief model for Brazilian Portuguese

**DOI:** 10.1590/1807-3107bor-2026.vol40.090

**Published:** 2026-11-09

**Authors:** Myrelle Leal Campos Sousa, Matheus de França Perazzo, Ramon Targino Firmino, Maria Clara Araújo do Nascimento, Colman Mcgrath, Rodrigo Franklin de Barros, Ana Flávia Granville-Garcia

**Affiliations:** a Universidade Estadual da Paraíba – UEPB, Postgraduate Program in Dentistry, Campina Grande, PB, Brazil.; b Universidade Federal de Goiás – UFG, School of Dentistry, Department of Social Dentistry, Goiânia, GO, Brazil.; c Universidade Federal de Campina Grande – UFCG, Patos, PB, Brazil.; d Universidade Estadual da Paraíba – UEPB, Graduate Program in Dentistry, Campina Grande, PB, Brazil.; e University of Hong Kong, School of Dentistry, Department of Dental Public Health, Hong Kong SAR, China.

**Keywords:** Adolescent Behavior, Dental Caries, Health Belief Model

## Abstract

This study aimed to culturally adapt and validate the Oral Health Behavior Questionnaire for Adolescents Based on the Health Belief Model (OHBQ) for Brazilian Portuguese. It was hypothesized that the Brazilian Portuguese version would demonstrate satisfactory psychometric properties and cultural appropriateness. The adaptation process included translation, synthesis, back-translation, independent expert review, pretesting, and expert committee review. The final version of the instrument was pretested in 30 adolescents and evaluated through cognitive interviews. The culturally adapted version (BR-OHBQ) was administered to 453 adolescents aged 12 to 17 years attending public and private schools in a city in northeastern Brazil. The psychometric properties evaluated included internal consistency (Cronbach's α), test-retest reliability (intraclass correlation coefficient [ICC]), convergent validity (correlation with the Oral Health Impact Profile-14 [OHIP-14]), predictive validity (associations with dental caries and visible plaque), and factorial validity (confirmatory factor analysis [CFA]). The BR-OHBQ demonstrated adequate cultural adaptation and satisfactory psychometric performance: α = 0.651 and ICC = 0.735 (Part A); α = 0.835 and ICC = 0.805 (Part B); and α = 0.861 and ICC = 0.711 (Part C). CFA demonstrated good model fit (χ² = 1267.547, df = 550; CFI = 0.903; RMSEA = 0.051, 95%CI: 0.047–0.055). No significant correlation was observed between the BR-OHBQ and OHIP-14 scores (*r*_s_ = −0.084, p > 0.05). However, correlations with dental caries (r_s_ = −0.134, p < 0.05) and visible plaque (r_s_ = −0.112, p < 0.05) were statistically significant. The BR-OHBQ was found to be a valid and reliable instrument for assessing beliefs related to oral health behaviors among Brazilian adolescents.

## Introduction

Social support, socioeconomic status, psychosocial characteristics, and oral health beliefs influence behavioral patterns among adolescents and predict oral hygiene quality in this population.^1^ Adolescents’ knowledge and beliefs regarding oral health are important determinants of behavioral change.^2,3^

The stability of positive oral health beliefs during adolescence promotes the maintenance of healthy oral habits into adulthood, reduces tooth loss due to dental caries, and improves the accuracy of self-assessments regarding oral hygiene.^4^ Conversely, negative beliefs may contribute to tooth loss, school absenteeism, and even bullying related to oral appearance.^5,6^

Parental cultural and psychosocial characteristics shape adolescents’ behavior and influence their oral health. The likelihood of toothbrushing at the recommended frequency increases nearly 50-fold when parents brush at least twice a day.^7,8^ Maternal education and perceived barriers predict adolescents’ attitudes, whereas high levels of social support and parents’ knowledge of available dental services reduce the prevalence of dental caries from childhood onward.^9^

Several instruments have been validated in Brazil to assess oral health-related quality of life among adolescents, including the Brazilian version of the Positive Oral Health and Well-Being (B-POHW) index, which assesses the potential to achieve and maintain oral health through positive oral health perceptions.^10^ The 14-item version of the Oral Health Impact Profile (OHIP-14) has also been validated and is widely used internationally to assess the impact of oral health status based on the perceptions of adults and adolescents.^11^

The Oral Health Behavior Questionnaire for Adolescents Based on the Health Belief Model (OHBQ) was developed to investigate adolescents’ oral health beliefs and behaviors within the framework of the Health Belief Model (HBM).^3^ For use in Brazil, the OHBQ requires cross-cultural adaptation followed by an evaluation of the psychometric properties of the translated and culturally adapted version.^12,13^

Therefore, the present study aimed to culturally adapt the OHBQ for Brazilian Portuguese-speaking adolescents and validate the Brazilian Portuguese version. It was hypothesized that the instrument would demonstrate cultural and linguistic equivalence and satisfactory psychometric properties and that higher scores would be associated with lower levels of visible plaque and lower caries experience among adolescents.

## Methods

### Ethical considerations

This study was approved by the Human Research Ethics Committee of Universidade Estadual da Paraíba (Approval no. 79632924.1.0000.5187). The participants’ legal guardians provided written informed consent, and the participants provided written assent prior to enrollment.

### Description of OHBQ

The HBM was developed to explain preventive health behaviors based on an individual's perceptions of susceptibility to and severity of health problems, which influence daily oral health practices and engagement in healthy behaviors.^14^ The OHBQ was originally developed in English by researchers in Hong Kong to assess oral health beliefs related to toothbrushing and dental floss use among adolescents aged 11 to 15 years. The instrument has been translated into Turkish and validated for use in Turkey.^15^ The questionnaire is self-administered, and its domains correspond to the six core constructs of the HBM. The OHBQ assesses perceived susceptibility (Items 1–2), perceived benefits (Items 3–9), perceived barriers (Items 10–15), cues to action (Items 16–18), perceived severity (Items 19–25), and self-efficacy (Items 26–35). The 35 items are organized into three parts and scored on a five-point Likert scale: Part A, ranging from 1 ("I strongly disagree") to 5 ("I strongly agree") for Items 1–18; Part B, ranging from 1 ("Not at all serious") to 5 ("Very serious") for Items 19–25; and Part C, ranging from 1 ("Not at all confident") to 5 ("Very confident") for Items 26–35. The maximum possible score is 175 points, with higher scores indicating stronger and more positive oral health beliefs and greater adoption of behaviors favorable to maintaining oral health.^3^

### Study design

A methodological validation study was conducted in two phases to validate the OHBQ for Brazilian Portuguese (BR-OHBQ). This study followed the *COnsensus-based Standards for the selection of health Measurement INstruments* (COSMIN) guidelines for validation studies.^16^ The first phase involved the cross-cultural adaptation of the questionnaire, and the second phase evaluated the psychometric properties of the BR-OHBQ.

### Study population

The target population comprised adolescents aged 12 to 17 years enrolled in public and private schools in the municipality of Campina Grande, Paraíba, Brazil.

### Sample size calculation

The minimum sample size was determined based on recommendations for instrument validation studies, which require two to 20 participants per questionnaire item and an absolute minimum of 100 to 250 participants.^17^ A minimum of 350 participants was established, with a 10% increase to compensate for potential dropouts, resulting in a final target sample of 385 participants.

### Eligibility criteria

Adolescents aged 12 to 17 years enrolled at public and private schools who had no systemic diseases or physical or learning disabilities, as reported by teachers, were eligible for inclusion. Non-native speakers of Brazilian Portuguese and adolescents undergoing orthodontic treatment were excluded. Initial contact with parents/guardians was established through the adolescents during the first school visit. After eligibility had been confirmed, the questionnaires were sent home to be completed by the parents/guardians.

### Cross-cultural adaptation

With permission from the authors of the original instrument, the cross-cultural adaptation for Brazilian Portuguese followed procedures to achieve conceptual, item, semantic, operational, measurement, and functional equivalence, based on established methodological guidelines.^12,18^ The process comprised the following steps:

- Initial translation: Two independent translators (a bilingual dentist and a translator with no prior knowledge of the healthcare field) independently translated the instrument into Brazilian Portuguese, emphasizing simple and easily understood language.
- Synthesis: The translators compared and synthesized the two forward translations into a single preliminary version. Any disagreements were resolved with the assistance of specialists in public health.
- Back-translation: Two native English-speaking translators with no prior knowledge of the instrument or the healthcare field independently translated the Brazilian Portuguese version back into English.
- Review and second version: The original authors and an expert committee compared the back-translated versions with the original instrument and produced a second synthesized version.
- Pretesting and cognitive interviews: The second synthesized Brazilian Portuguese version was pretested in 30 adolescents. Cognitive interviews using a qualitative approach were conducted to identify and minimize potential sources of bias.^16^
- Final review and final version: The expert committee reviewed all suggestions and, because no further modifications were considered necessary, approved the final version of the instrument for psychometric evaluation.

### Calibration

Examiner agreement was assessed using Cohen's kappa statistic.^19^ Examiner calibration for dental caries detection was performed using the International Caries Detection and Assessment System (ICDAS).^20^ Visible plaque was assessed using the Visible Plaque Index (VPI) and dichotomized as present or absent for each sextant (upper right, upper anterior, upper left, lower right, lower anterior, and lower left).^21^ Training consisted of a theoretical phase conducted by the gold-standard examiner for the diagnostic indices, followed by a practical calibration phase and reassessment after seven days. Intra- and inter-examiner reliability yielded kappa values ≥ 0.81, confirming excellent examiner agreement.^19^

### Pilot study

Prior to the main study, a pilot study involving 20 participants was conducted to evaluate the proposed methodology and the feasibility of the data collection procedures, including questionnaire administration. The pilot study was conducted in person at a public school selected for convenience. Participants enrolled in the pilot study were not included in the main study.

### Non-clinical data collection

Parents/guardians completed a sociodemographic questionnaire that collected information about themselves (sex, age, relationship to the adolescent, educational attainment, and income) and about the adolescent, including dental attendance history and frequency of visits (none, once, or two or more times), reason for the dental visit (preventive care, treatment, or toothache), and previous experience of toothache.

The 14-item Oral Health Impact Profile (OHIP-14) was used to assess perceived oral health-related quality of life during the previous six months using a five-point scale ranging from 0 (never) to 4 (always),^22^ with higher scores indicating greater negative impact on oral health-related quality of life. The mean OHIP-14 score of each adolescent-parent/guardian pair was used to assess convergent validity by examining its correlation with the BR-OHBQ score.

### Clinical data collection

Data were collected from August to December 2024. Clinical examinations were performed after distribution of oral hygiene kits and supervised toothbrushing. Fluoride gel was applied to adolescents with active dental caries. Dental caries was diagnosed using the ICDAS. Codes 2 (visible white spot without drying) through 6 (extensive cavity with visible dentin) were considered indicative of caries in the present study.^20^ Visible plaque was assessed using the VPI and classified as present or absent for each of the six sextants of the mouth.^21^

### Data processing and analysis

To assess temporal stability, the questionnaire was re-administered to 20% of the sample after 15 days. The reliability of the BR-OHBQ was evaluated by internal consistency using Cronbach's alpha (α) and McDonald's omega (ω), as well as by test-retest reliability using the intraclass correlation coefficient (ICC), with 95% confidence intervals. Values of α and ω were classified as high (≥ 0.90), acceptable (0.70–0.89), borderline (0.60–0.69), or low (< 0.60).^13,16,18,23^ Structural validity was investigated using confirmatory factor analysis (CFA), based on the chi-square statistic (χ²), degrees of freedom (df), comparative fit index (CFI), and root mean square error of approximation (RMSEA). Reference values indicative of adequate model fit were χ²/df < 2, CFI ≥ 0.95, and RMSEA ≤ 0.06.^24,25^ Moreover, the Kaiser-Meyer-Olkin (KMO) measure and Bartlett's test of sphericity were used to determine the suitability of the data for factor analysis.

Construct validity comprised a) convergent validity, assessed using Spearman's correlation coefficients between the BR-OHBQ and OHIP-14 scores, and b) predictive validity, assessed using Spearman's correlation coefficients between the total BR-OHBQ score and the clinical variables (visible plaque, dental caries, anterior dental caries, and visible plaque in the sextants), with statistical significance set at *p* < 0.05. Statistical analyses were performed using IBM SPSS version 22.0 and JASP version 0.19.3.

## Results

### Adaptation to Brazilian Portuguese (BR-OHBQ)

The final sample comprised 453 adolescents aged 12 to 17 years enrolled in public and private schools in the city of Campina Grande, Brazil. The pretest, conducted with 30 adolescents aged 12 to 17 years (both sexes), the majority of whom were male (60%), with a mean age of 14.43 years, confirmed the clarity and comprehensibility of the BR-OHBQ. Cohen's kappa values ranged from 0.88 (inter-examiner agreement) to 0.89 (intra-examiner agreement), indicating excellent examiner agreement. Participants completed the questionnaire under supervision, and cognitive interviews confirmed that all items were clearly understood, with no need for revisions. Accordingly, no structural or linguistic modifications were required, and the final Brazilian Portuguese version was submitted to the original authors for approval.

### Characterization of the sample

The final sample comprised 453 adolescents aged 12 to 17 years (mean age: 13.39 ± 1.29 years). The sex distribution was balanced, with a slight predominance of males (53%). Most participants attended public schools (74.2%), were enrolled in the morning shift (82.6%), and presented with visible plaque in at least one sextant (82.8%). Most participants had no caries experience in anterior teeth (64.5%), and the prevalence of tooth loss due to caries was 6.4%.

The mean age of the parents/guardians was 40.38 ± 8.9 years. Most parents/guardians were women (72.1%), the majority of whom were mothers (72%). Completion of high school was the most common educational attainment (24.8%), and a monthly family income of up to one minimum wage was the most prevalent income category (47.5%). Most adolescents reported a previous history of toothache (60.4%), and 46% had sought dental care for treatment. The mean number of dental visits during the previous year was 1.0 ± 0.05.

BR-OHBQ scores ranged from 38 to 146, with a mean total score of 104.24 ± 15.78. Score ranges were 18–71 for Part A, 7–35 for Part B, and 10–50 for Part C. The mean OHIP-14 score was 11.79 ± 9.47 (range: 0–48) for adolescents and 11.66 ± 9.25 (range: 0–50) for parents/guardians (Table 1).

**Table 1:** Characteristics of the sample (n = 453). Campina Grande, PB, Brazil, 2024.

Variables		Frequency (n)	Percentage (%)
Adolescents’ sex			
	Male	240	53
	Female	212	46.8
Type of school			
	Public	336	74.2
	Private	117	25.8
Class hours			
	Morning	374	82.6
	Afternoon	79	17.4
Parents’/guardians’ sex			
	Male	58	20.9
	Female	217	72.1
Relationship to adolescent			
	Mother	202	72.2
	Father	43	15.5
	Brother	11	4.0
	Uncle/aunt	6	2.2
	Grandparent	7	2.5
	Other	2	.7
Guardian's schooling			
	Did not study	2	.7
	1^st^ to 4^th^ grade incomplete	13	4.7
	1^st^ to 4^th^ grade complete	5	1.8
	5^th^ to 8^th^ grade incomplete	29	10.4
	5^th^ to 8^th^ grade complete	23	8.3
	High school incomplete	35	12.6
	High school complete	69	24.8
	Higher education incomplete	44	15.8
	Higher education complete	42	15.1
Monthly family income*			
	No income	19	6.8
	Up to R$1,412	132	47.5
	Up to R$2,824	76	27.3
	Up to R$4,236	30	10.8
	Up to R$7,060	7	2.5
	Up to R$14,120	1	0.4
	Up to R$28,240	6	2.2
	More than R$28,240	2	0.7
History of dental visits			
	Yes	227	81.7
	No	47	16.9
History of toothache			
	Yes	168	60.4
	No	106	38.1
Reason for visit			
	Prevention	65	23.4
	Treatment	128	46.0
	Toothache	35	12.6
Mean age – adolescents		13.39 ± 1.29	
Mean age - parents/guardians		40.38 ± 8.95	
Mean BR-OHBQ score		104.24 ± 15.70	
Mean OHIP-14 score - adolescents		11.79 ± 9.47	
Mean OHIP-14 score - parents/guardians		11.66 ± 9.25	
Mean number of visits in the previous year		1.13 ± 0.05	

Totals may vary because of missing responses.

* Monthly family income included wages, family grants, unemployment insurance, and income from odd jobs.

### Brazilian version of the OHBQ (BR-OHBQ)

Based on the model goodness-of-fit analysis and CFA parameters, the BR-OHBQ did not require any structural modifications. The instrument retained its 35 items, scored on a five-point Likert scale ranging from 1 ("I strongly disagree") to 5 ("I strongly agree") for Items 1–18; from 1 ("Not at all serious") to 5 ("Very serious") for Items 19–25, and from 1 ("Not at all confident") to 5 ("Very confident") for Items 26–35 (Table 2).

**Table 2:** Questionnaire items in the original and cross-culturally adapted Brazilian Portuguese (BR-OHBQ) versions.

Item	Original version					Brazilian version (BR-OHBQ)				
**Part A**										
*Strongly agree*			Agree		Neutral		Disagree		Strongly disagree	
**Concordo totalmente**			**Concordo em parte**		**Nem concordo nem discordo**		**Discordo em parte**		**Discordo totalmente**	
**1**	There is a chance that I will get caries.					Existe uma chance de que eu vou ter cárie.				
**2**	There is a chance that I will have periodontal disease.					Existe uma chance de que eu vou ter sangramento na gengiva.				
**3**	I think brushing and flossing can make teeth healthier.					Escovar os dentes e usar fio dental pode deixar os dentes mais saudáveis.				
**4**	I think brushing and flossing can prevent oral diseases from happening.					Escovar os dentes e usar fio dental pode evitar cárie e sangramento na gengiva.				
**5**	I think brushing and flossing can make teeth look good.					Escovar os dentes e usar fio dental pode deixar os dentes bonitos.				
**6**	I think brushing and flossing can keep breath fresh.					Escovar os dentes e usar fio dental pode manter um bom hálito.				
**7**	I think brushing and flossing can prevent inconvenient eating caused by oral diseases.					Escovar os dentes e passar fio dental pode evitar problemas para comer causados por cárie e sangramento na gengiva.				
**8**	I think brushing and flossing can help me avoid spending more time on dental treatment in the future.					Escovar os dentes e passar fio dental pode evitar tratamentos mais longos nos dentes.				
**9**	I think brushing and flossing can help me avoid spending more money on dental treatment in the future.					Escovar os dentes e passar fio dental pode me ajudar a economizar mais dinheiro em tratamento nos dentes.				
**10**	I think it is difficult for me to brush twice a day.					É difícil escovar os dentes duas vezes por dia.				
**11**	I think it's a waste of time to brush and floss.					É perda de tempo escovar os dentes e usar fio dental.				
**12**	I think I do not have enough time to have an annual dental visit.					Eu não tenho tempo para ir ao dentista uma vez por ano.				
**13**	I am afraid of undergoing tooth treatment, so I don't have dental visits annually.					Eu não vou ao dentista todo ano porque eu tenho medo de tratar os dentes.				
**14**	I think we have no money at my home, so I don't have an annual dental visit.					Eu não vou ao dentista todo ano por falta de dinheiro em casa.				
**15**	I think the dental clinic is far from my home, so I don't have an annual dental visit.					Eu não vou ao dentista todo ano porque fica longe da minha casa.				
**16**	Parents often reminds me of brushing and flossing.					Meus pais me lembram com frequência de escovar os dentes e usar fio dental.				
**17**	Classmates often reminds me of brushing and flossing.					Meus colegas me lembram com frequência de escovar os dentes e usar fio dental.				
**18**	Teachers often reminds me of brushing and flossing.					Meus professores me lembram com frequência de escovar os dentes e usar fio dental.				
**Part B**										
Not at all serious			A little serious		Partially serious		Serious		Very serious	
**Não é grave**			**É um pouco grave**		**É mais ou menos grave**		**É grave**		**É muito grave**	
**19**	If I have caries, for me that is…					Se eu tiver cárie, para mim isso…				
**20**	If I have periodontal disease, for me that is…					Se eu tiver sangramento na gengiva, para mim isso…				
**21**	If my teeth do not look good because of oral diseases, for me that is…					Se meus dentes não estiverem bonitos por causa da cárie e do sangramento na gengiva, para mim…				
**22**	If I have bad breath because of oral diseases, for me that is…					Se eu tiver mau hálito por causa da cárie e do sangramento na gengiva, para mim isso…				
**23**	If I can't sleep well because of oral diseases, for me that is…					Se eu não conseguir dormir bem por causa da cárie e do sangramento na gengiva, para mim isso…				
**24**	If I can't eat my favorite food because of oral diseases, for me that is…					Se eu não conseguir comer minha comida favorita por causa da cárie e do sangramento na gengiva, para mim isso…				
**25**	If I get laughed at by classmates because of oral diseases, for me that is…					Se meus colegas rirem de mim por causa da cárie e do sangramento na gengiva, para mim isso…				
**Part C**										
How confident you are that you will brush your teeth for 2 min twice daily on the circumstances below?										
Qual é sua certeza de escovar os dentes duas vezes ao dia durante dois minutos em cada uma das situações abaixo?										
Not confident			A bit confident		Fairly confident		Quite confident		Very confident	
	**Não tenho certeza**		**Tenho pouca certeza**		**Tenho mais ou menos certeza**		**Tenho muita certeza**		**Tenho muitíssima certeza**	
**26**	When you are under a lot of stress					Quando eu estou muito estressado				
**27**	During or after experiencing personal problems					Durante ou depois de passar por problemas pessoais				
**28**	When you are feeling tired					Quando eu estou cansado				
**29**	When you don't feel like it					Quando eu estou sem vontade				
**30**	When you are anxious					Quando estou ansioso				
**31**	After experiencing family problems					Depois de passar por problemas na família				
**32**	When you have other commitments					Quando eu tenho outros compromissos				
**33**	When you feel you don't have the time					Quando eu acho que não tenho tempo				
**34**	When you are feeling under pressure from school work					Quando eu me sinto pressionado com as tarefas da escola				
**35**	When you have too much work to do at home					Quando eu tenho muito serviço de casa para fazer				

The results indicated that the items were organized consistently with the original theoretical framework, with adequate factor loadings and no significant overlap among factors. The original three-part structure was maintained: Part B corresponds to perceived severity, Part C to self-efficacy, and Part A encompasses the remaining HBM constructs.

CFA supported the three-factor structure of the instrument. The fit indices were within acceptable limits, indicating good model fit to the Brazilian data: χ² = 1267.547 df = 550; CFI = 0.903; RMSEA = 0.051 (95%CI: 0.047–0.055); KMO = 0.72252; and Bartlett's test of sphericity: χ² = 5059.9, df = 595; p < 0.001).

The BR-OHBQ demonstrated borderline internal consistency for Part A (α = 0.651; ω = 0.496; ICC = 0.735) and acceptable to high internal consistency for Parts B (α = 0.835; ω = 0.838; ICC = 0.805) and C (α = 0.861; ω = 0.864; ICC = 0.711), with acceptable test-retest reliability across all three parts. No significant correlation was observed between the BR-OHBQ and OHIP-14 scores (r_s_ = −0.084, p > 0.05). BR-OHBQ scores were negatively correlated with the ICDAS (r_s_ = −0.134, p < 0.05) and VPI (r_s_ = −0.112, p < 0.05), indicating significant associations with caries in the anterior teeth and visible plaque in at least one sextant of the mouth, thereby supporting the predictive validity of the instrument.

## Discussion

This study culturally adapted and validated the OHBQ for use in Brazil while preserving its original multidimensional structure and demonstrating satisfactory structural validity despite heterogeneous psychometric performance across domains. Higher scores reflected stronger oral health beliefs associated with healthier oral hygiene behaviors. The instrument demonstrated acceptable overall reliability, with adequate internal consistency for the total scale and for Parts B and C. Test–retest reliability and item–total correlations further supported its stability and internal coherence.^26^ These findings are consistent with those reported for the original instrument and a subsequent validation study.^3,15^

Convergent validity with the OHIP-14 was not confirmed, likely because the two instruments assess complementary but distinct dimensions of oral health. The BR-OHBQ evaluates beliefs and behavioral determinants, whereas the OHIP-14 assesses the perceived impact of oral health on quality of life.^3,11^ The wide score range indicates adequate discriminative ability,^16,18^ and broad score distributions across populations from different cultural contexts have also been reported without compromising data validity.^15^

Lower BR-OHBQ scores were associated with the presence of caries in the anterior teeth and visible plaque in at least one sextant of the mouth. The occurrence of anterior dental caries in more than one-third of the sample suggests esthetic and functional impairment with potential psychosocial consequences. Untreated dental caries is the principal oral health predictor of bullying during adolescence.^27^ Low income and limited educational attainment also increase exposure to risk factors and may intensify the negative effects of conditions affecting the physical, psychological, and social well-being of adolescents.^28^

Visible plaque is a direct marker of risk for dental caries and periodontal disease, and its high prevalence suggests inadequate oral hygiene practices. Previous studies have shown that socioeconomic factors and toothbrushing frequency are associated with plaque levels among adolescents.^1,29^ Toothbrushing technique and the uniform distribution of brushing time may serve as indicators of long-term oral hygiene quality, as reflected by clinical plaque and gingival indices.^30^

Despite showing the expected inverse direction, the weak correlations observed in this study highlight that oral health outcomes depend on complex socio-environmental and biological determinants rather than isolated perceptions.^1,28^ These findings reinforce the role of the BR-OHBQ as a complementary behavioral measure rather than a direct clinical predictor.^3,4,15^

Greater recognition of the importance of oral health among parents/guardians encourages the maintenance of regular toothbrushing habits in both themselves and their children.^8^ Most respondents in the present study were mothers and had low income and a high school education. Parents with low educational attainment have a poorer understanding of the severity of dental caries and the benefits of recommended oral health behaviors, in addition to lower confidence in their ability to maintain these behaviors.^31^

BR-OHBQ scores were not correlated with OHIP-14 scores. As reported previously, age was not associated with perceived oral health–related quality of life.^32^ However, parental modeling influences adolescents’ behavioral patterns, and positive oral health beliefs among parents/guardians increase toothbrushing frequency in adolescents.^8^ Low parental educational attainment increases perceived barriers and reinforces the belief that control over their children's oral health depends on the dentist or on chance.^31^

Lower scores on the factors comprising Part A tend to indicate lower externality. Individuals with lower perceived susceptibility to health problems are less responsive to internal and external cues to adopt recommended health behaviors.^14^ Psychological interventions that take these factors into account have beneficial effects on the oral health of children and adolescents.^33^ Moreover, positive oral health behaviors (such as flossing) and stronger health beliefs are associated with greater willingness to attend regular dental checkups.^4^

The perceived severity and self-efficacy subscales exhibited excellent factor loadings in the BR-OHBQ. Therefore, higher scores on Parts B and C may indicate stronger perceptions of these constructs. Socioeconomic vulnerability, limited social support, and low resilience increase the likelihood of unhealthy behaviors and negatively affect adolescents’ quality of life.^1^ Stronger internal health beliefs and greater self-efficacy favor a collaborative and empowering dentist-patient relationship,^34^ whereas negative beliefs increase susceptibility to harmful oral health behaviors.^3^

The experience of traditional bullying victimization during childhood may also increase susceptibility to internalizing and externalizing problems.^35^ The adolescents in the present study had a mean age of 13 years and attended both public and private schools. This population may be particularly vulnerable to both bullying and the influence of social support on their self-esteem and quality of life. Positive self-esteem during adolescence has been associated with improved oral health-related quality of life and a predominance of beneficial oral health beliefs and behaviors, including reduced sugar consumption.^1^

The present study validated the BR-OHBQ as a reliable instrument with adequate structural validity for assessing oral health beliefs among Brazilian adolescents. The instrument assesses cognitive factors that influence oral health outcomes and preventive practices, thereby supporting the development of effective oral health promotion interventions.^33^ Extending the target age range to 12–17 years reflects the increasing autonomy of adolescence and aligns with school-based research while preserving the satisfactory psychometric properties of the instrument and stable factor structure.^13,23,25^

**Figure:**
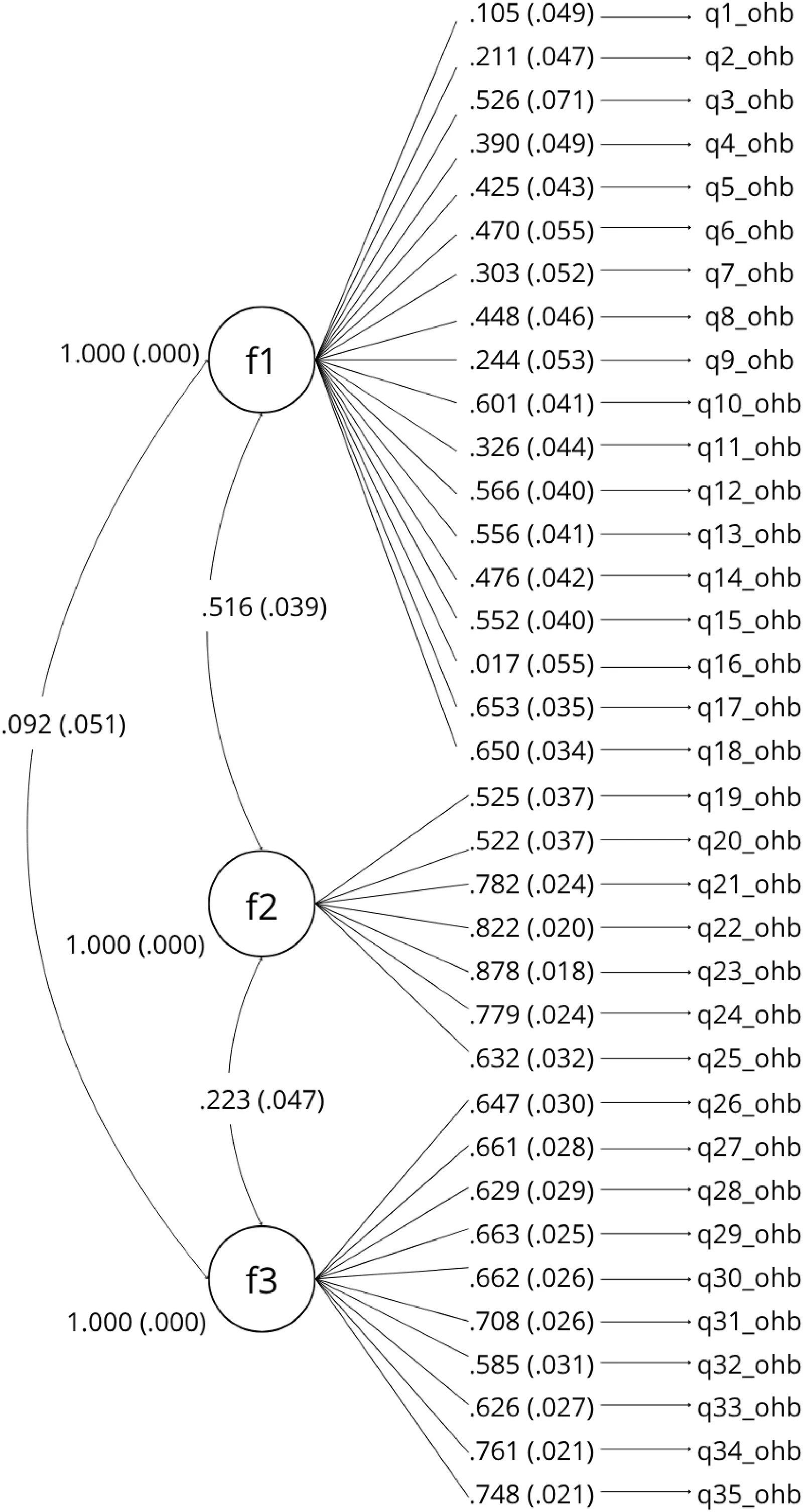
Fitness of model and CFA parameters of Brazilian version of OHBQ (BR-OHBQ).

This study has some limitations. Comparative studies evaluating personal beliefs, oral hygiene, and oral health outcomes are scarce. Moreover, the study was conducted in a single geographic region. Therefore, further qualitative studies and additional validation in different regions and sociocultural contexts are warranted.

## Conclusion

The cross-cultural adaptation and validation of the OHBQ for Brazilian Portuguese (BR-OHBQ) demonstrated that the instrument is a psychometrically valid and reliable measure for assessing oral health beliefs related to oral health behaviors among Brazilian adolescents. Its use may support clinical practice by identifying cognitive factors associated with oral health and guiding interventions aimed at promoting healthier oral behaviors.

## Data Availability

The datasets generated during and/or analyzed during the current study are available from the corresponding author on reasonable request.
